# Comparison of Liver Cell Models Using the Basel Phenotyping Cocktail

**DOI:** 10.3389/fphar.2016.00443

**Published:** 2016-11-21

**Authors:** Benjamin Berger, Massimiliano Donzelli, Swarna Maseneni, Franziska Boess, Adrian Roth, Stephan Krähenbühl, Manuel Haschke

**Affiliations:** ^1^Division of Clinical Pharmacology and Toxicology, University Hospital BaselBasel, Switzerland; ^2^Department of Biomedicine, University of BaselBasel, Switzerland; ^3^Roche Innovation Center Basel, Pharmaceutical Sciences, Hoffmann-La Roche Ltd.Basel, Switzerland; ^4^Swiss Center for Applied Human ToxicologyBasel, Switzerland

**Keywords:** cytochrome P450 (CYP), basel cocktail, 3-dimensional spheroid primary human hepatocyte culture, CYP induction, liquid chromatography tandem mass spectrometry

## Abstract

Currently used hepatocyte cell systems for *in vitro* assessment of drug metabolism include hepatoma cell lines and primary human hepatocyte (PHH) cultures. We investigated the suitability of the validated *in vivo* Basel phenotyping cocktail (caffeine [CYP1A2], efavirenz [CYP2B6], losartan [CYP2C9], omeprazole [CYP2C19], metoprolol [CYP2D6], midazolam [CYP3A4]) *in vitro* and characterized four hepatocyte cell systems (HepG2 cells, HepaRG cells, and primary cryopreserved human hepatocytes in 2-dimensional [2D] culture or in 3D-spheroid co-culture) regarding basal metabolism and CYP inducibility. Under non-induced conditions, all CYP activities could be determined in 3D-PHH, CYP2B6, CYP2C19, CYP2D6, and CYP3A4 in 2D-PHH and HepaRG, and CYP2C19 and CYP3A4 in HepG2 cells. The highest non-induced CYP activities were observed in 3D-PHH and HepaRG cells. mRNA expression was at least four-fold higher for all CYPs in 3D-PHH compared to the other cell systems. After treatment with 20 μM rifampicin, mRNA increased 3- to 50-fold for all CYPs except CYP1A2 and 2D6 for HepaRG and 3D-PHH, 4-fold (CYP2B6) and 17-fold (CYP3A4) for 2D-PHH and four-fold (CYP3A4) for HepG2. In 3D-PHH at least a two-fold increase in CYP activity was observed for all inducible CYP isoforms while CYP1A2 and CYP2C9 activity did not increase in 2D-PHH and HepaRG. CYP inducibility assessed *in vivo* using the same phenotyping probes was also best reflected by the 3D-PHH model. Our studies show that 3D-PHH and (with some limitations) HepaRG are suitable cell systems for assessing drug metabolism and CYP induction *in vitro*. HepG2 cells are less suited to assess CYP induction of the 2C and 3A family. The Basel phenotyping cocktail is suitable for the assessment of CYP activity and induction also *in vitro*.

## Introduction

Cytochrome P450 enzymes (CYPs) are involved in the oxidative metabolism of the majority of the commonly used low molecular weight drugs, thereby influencing the pharmacokinetics of these drugs and having an important role in drug-drug interactions (Wilkinson, 2005). Genetic variants and exogenous factors such as diet, smoking habits, and concomitant medication can affect the activity of CYPs. All of these factors are responsible for wide interindividual variations in CYP activity and drug plasma concentrations, which can be associated with either increased toxicity or an insufficient pharmacological effect.

Simultaneous determination of the activity of different CYPs in an individual patient can be performed by administering a combination (“cocktail”) of specific probe drugs (Fuhr et al., 2007). Several cocktails have been characterized in clinical studies and are used in individual patients, including the Karolinska cocktail (Christensen et al., 2003), the Cooperstown 5+1 cocktail (Chainuvati et al., 2003), the Geneva cocktail (Bosilkovska et al., 2014) and the Basel Cocktail (Donzelli et al., 2014). These cocktails have also been shown to be valuable tools to assess the capacity of drugs to induce or inhibit CYPs (Bosilkovska et al., 2014; Derungs et al., 2016). Drug cocktails have also been used to perform *in vitro* studies, in particular when assessing the CYP inhibition and induction potential of chemical compounds (Youdim et al., 2007; Mori et al., 2009; Spaggiari et al., 2014).

Suitable *in vitro* systems to assess drug metabolism are mainly liver microsomes, hepatocarcinoma cell cultures (e.g., HepG2 and HepaRG cells) and primary human hepatocytes (PHHs). Monolayer-grown PHHs or hepatocarcinoma cell-lines are currently the gold standard for *in vitro* drug-drug interaction and hepatotoxicity studies. However, monolayer hepatocyte culture systems have major drawbacks. PHH cultures suffer from a rapid loss of cell polarity and of specific hepatic functions, which limits their applicability for drug metabolism experiments to a few days. Furthermore, they exhibit a large variation in cell functions, especially in CYP activities, as well as a variable response to CYP inducers (Abadie-Viollon et al., 2010; Godoy et al., 2013). In comparison, hepatocarcinoma cell line cultures can be used over extended periods of time, but their metabolic activity and response to CYP inducers are generally limited (Donato and Castell, 2003). As an alternative to 2D cultures of PHHs or hepatocarcinoma cell lines, a variety of different 3D liver models are being explored, as they are thought to approximate the *in vivo* tissue structure and cell behavior more closely (Godoy et al., 2013; Roth and Singer, 2014).

Limited availability of phenotyping probe drugs is a frequent limitation of published *in vivo* phenotyping cocktails. To facilitate clinical application of phenotyping, we therefore developed a new low-dose cocktail, which is based on probe drugs that are widely used in clinical practice (Donzelli et al., 2014). In a subsequent study, we characterized this cocktail in healthy male subjects treated with a combination of different CYP inhibitors and a CYP inducer (Derungs et al., 2016). Since we now have a detailed *in vivo* characterization of this cocktail, it was of interest to also test the cocktail *in vitro*. The two principle aims of our investigation were therefore (i) to assess the performance and usability of the Basel cocktail *in vitro* by comparing *in vitro* with *in vivo* results and (ii) to assess the suitability of different hepatocyte cell models for the investigation of drug metabolism and CYP induction.

In order to accomplish these aims, we tested the metabolism of the Basel cocktail (containing caffeine, metoprolol, omeprazole, losartan, efavirenz, and midazolam) in HepG2 cells, HepaRG cells, and primary cryopreserved human hepatocytes grown in 2D and in 3D culture under basal conditions and after CYP induction with rifampicin. CYP induction achieved by rifampicin *in vitro* could then be compared with the extent of *in vivo* CYP induction.

## Materials and methods

### Chemicals and reagents

8′-hydroxyefavirenz, efavirenz-d4, losartan, losartan-carboxylic acid (E3174), losartan-d4, omeprazole, 5′-hydroxyomeprazole, omeprazole-d3, metoprolol, α-hydroxymetoprolol, and metoprolol-d7 were purchased from TRC (Toronto, Canada). 1′-hydroxymidazolam and midazolam-d6 were acquired from Lipomed (Arlesheim, Switzerland), whereas rifampicin and β-glucuronidase (type HP-2 from *Helix pomatia*) were obtained from Sigma-Aldrich (Sigma- Aldrich Chemie GmbH, Buchs, Switzerland). Midazolam (F. Hoffmann-La Roche, Basel, Switzerland) and efavirenz (Merck, NJ, USA) were kindly provided by the respective manufacturers. The chemical structures of the probe drugs and their phase I metabolites are provided in Supplementary Figure 2. Formic acid, HPLC grade methanol, and HPLC grade water were purchased from Merck (Darmstadt, Germany). Media used were purchased from GIBCO (Lucerne, Switzerland).

Stock solutions, calibration, and quality control spiking solutions were prepared in DMSO. Calibration standards and quality controls were prepared by enriching the respective medium with the corresponding spiking solutions. Internal standard solutions containing the deuterated cocktail probe drugs were prepared in methanol.

### Cell cultures

The human hepatoma cell line HepG2 was obtained from ATCC (Manassas, VA, USA). HepG2 cells were cultured in Dulbecco's modified Eagle's medium (DMEM; with 2 mM GlutaMAX®, 1.0 g/l glucose and sodium bicarbonate) supplemented with 10% (v/v) heat-inactivated fetal calf serum (FCS), and 10 mM HEPES buffer, pH 7.2. In general, all cells were kept at 37°C in a humidified 5% CO2 cell culture incubator and passaged using trypsin, while the cell number was determined using a Neubauer hemacytometer. Viability was checked using the tryptan blue exclusion method. For the experiments, HepG2 cells were seeded at 10,000 cells/well in 96-well plates or 150,000 cells/well in 12-well plates.

HepaRG cells were purchased from Biopredic International (Rennes, France) as undifferentiated cryopreserved cells with the associated medium. Freshly split HepaRG cells were seeded at 9000 cells/well in 96-well plates and treated over the course of the next 4 weeks as previously described (Aninat et al., 2006).

Primary cryopreserved human hepatocytes (Life Technologies, Lot Hu8119, female donor) were plated at 50,000 cells/ well in collagen type-1 precoated 96-well plates in William's E medium supplemented with 10% FBS (v/v), 1% L-Glutamine 200 mM (v/v), 1% Pen Strep (v/v), 0.1% dexamethasone 100 μM (v/v), and 0.1% insulin 100 μM (v/v) and left to incubate at 37°C, 95% humidity, 5% CO_2_.

For the 3D-hepatocyte co-culture model originally described by Ohkura et al. (2014), 3T3 fibroblast cells from Swiss albino mouse embryo tissue (3T3 Balb/clone A31, ATCC product number CCL 163) were seeded at a density of 8000 cells/well in 96-well plates or at 40,000 cells/well in 24-well plates into micro-patterned plates (Cell-able®, Cosmo Bio USA Inc., CA, USA) in Dulbecco's Modified Eagle's medium (DMEM; with 2 mM GlutaMAX®), supplemented with 10% FBS. The cells were cultured in a 5% CO_2_, 95% air humidified environment at 37°C. After 2 days, cryopreserved human hepatocytes (Life Technologies Lot Hu8119) were seeded at a density of 25,000 cells/well in the same 96-well plates containing the 3T3 cells (or 125,000 cells/well in 24-well plates). The 3T3-cryopreserved human hepatocyte co-culture was maintained for a minimum of 2 days to allow for spheroid formation, using the same medium as for the conventional two-dimensional (2D) cryopreserved hepatocyte culture.

Cryopreserved human hepatocyte preparations were only used for experiments if their morphological characteristics and viability (>85%) were acceptable.

### Markers of CYP450 activity

CYP activity was assessed by the addition of a CYP probe drug cocktail consisting of caffeine (CYP1A2, 80 μM), efavirenz (CYP2B6, 10 μM), losartan (CYP2C9, 14 μM), omeprazole (CYP2C19, 17 μM), metoprolol (CYP2D6, 23 μM), and midazolam (CYP3A4, 5 μM). The substrates were used at concentrations close to their K_*m*_ values previously published.

A previously developed liquid chromatography tandem mass spectrometry (LC-MS/MS) method (Donzelli et al., 2014) was used to analyze the phase I metabolites of the probe drugs. Chromatographic separation was performed on a Shimadzu HPLC system (Shimadzu AG, Reinach, Switzerland) coupled to a triple quadrupole tandem mass spectrometer (API4000, AB/MDS Sciex, Concord, Canada) operating in positive electrospray ionization mode, except for 8′-hydroxyefavirenz, which was detected in negative mode. Total run time was 2.9 min. Inter-assay accuracy (determined as the % bias) ranged from −10.7 to 9.8 and inter-assay precision (determined as the CV%) was lower than 11.3 for all analytes. The lower limit of quantification (LLOQ) was 0.25 ng/ml for hydroxymetoprolol, 8-hydroxyefavirenz, 1′-hydroxymidazolam, 5-hydroxyomeprazole, E-3174, and 0.5 ng/ml for paraxanthine.

### Cyp450 induction experiments

#### Evaluation of mRNA expression

HepG2 cells, HepaRG cells, 2D-cultured human hepatocytes, and 3D-cultured human hepatocytes were seeded in 24-well plates and treated for 48 h with rifampicin 20 μM. Three hundred and fifty microliters of RLT buffer (Qiagen, Hombrechtikon, Switzerland) was used to lyse the respective hepatocytes, after which the lysate was transferred to Qiashredder columns and centrifuged for 2 min at 13,000 rpm. From the eluate, total RNA was extracted and purified according to the manufacturer's instructions (Qiagen, RNeasy mini extraction kit). The concentration of the extracted RNA was measured spectrophotometrically at 260 nm on a NanoDrop 2000 (Thermo Fisher Scientific, Wohlen, Switzerland). cDNA was reverse-transcribed from the isolated RNA using the Qiagen omniscript system. For quantitative RT-PCR 10 ng cDNA was used. Forward and reverse primers for all CYPs tested and endogenous references, hypoxanthine phosphoribosyltransferase 1 (HPRT1) and Glyceraldehyde 3-phosphate dehydrogenase (GAPDH), were purchased from Microsynth (Balgach, Switzerland; listed in Table 1). RT-PCR was performed using SYBR green (Roche Diagnostics, Rotkreuz, Switzerland) on an ABI PRISM 7700 sequence detector (PE Biosystems, Rotkreuz, Switzerland). Quantification of mRNA expression levels was performed using the comparative-threshold cycle method (Livak and Schmittgen, 2001).

**Table 1:** **Gene-specific primers for RT-PCR**.

**Target gene**	**Organism**		**Primer Sequence (5′–3′)**	**Length (bp)**
CYP1A2	Human	Fw	GGACAGCACTTCCCTGAGAG	20
		Rev	GCTCCTGGACTGTTTTCTGC	20
CYP2B6	Human	Fw	CAGTGAATTCAGCCACCAGA	20
		Rev	ATTTTGGCTCGGTCATGAAG	20
CYP2C9	Human	Fw	AGGAAAACGGATTTGTGTGG	20
		Rev	GGCCATCTGCTCTTCTTCAG	20
CYP2C19	Human	Fw	GGATTGTAAGCACCCCCTG	19
		Rev	TAAAGTCCCGAGGGTTGTTG	20
CYP2D6	Human	Fw	TGTGCCCATCACCCAGAT	18
		Rev	AAGGTGGAGACGGAGAAGC	19
CYP3A4	Human	Fw	TACACAAAAGCACCGAGTGG	20
		Rev	TGCAGTTTCTGCTGGACATC	20
HPRT1	Human	Fw	GGTCCTTTTCACCAGCAAGCT	21
		Rev	TGACACTGGCAAAACAATGCA	21
GAPDH	Human	Fw	AGCCACATCGCTCAGACAC	19
		Rev	GCCCAATACGACCAAATCC	19

#### Functional assessment of CYP induction

HepG2 cells, HepaRG cells, cryopreserved human hepatocytes, as well as three-dimensionally seeded co-cultured cryopreserved human hepatocyte spheroids, were cultured in a 5% CO_2_ and 95% air humidified atmosphere at 37°C. Induction treatment (rifampicin 20 μM) lasted for 72 h, with the medium being changed every 24 h. Rifampicin stock solution was prepared in DMSO and further diluted in the appropriate culture medium to achieve a final DMSO concentration of 0.1% (v/v). Experimental control culture wells were treated with solvent [DMSO, 0.1% (v/v)] alone. Following induction treatment, CYP activity was assessed by the addition of fresh medium containing the cocktail probe drugs to the control and pre-treated cells. Substrates were dissolved and serially diluted in DMSO to the required concentrations. The final concentration of DMSO during the cocktail incubation was 0.2% (v/v). At selected time points (0, 15, 30, 45, 60, 90, and 120 min) the incubation was stopped by the addition of a threefold volume of ice-cold methanol containing the respective internal standards. The bottom of the wells was scraped using a pipette tip, after which the contents were transferred to an autosampler vial. After vigorous shaking (10 min) and centrifugation (3220 g, 30 min, 10°C) the supernatants were stored at −20°C until quantification by LC-MS/MS.

To determine the velocity of midazolam 1′-hydroxylation and efavirenz 8′-hydroxylation, the entire content of the autosampler vials were evaporated using a minivap microplate evaporator (Porvair Sciences Ltd., King's Lynn, Norfolk, UK). The analytes were then resuspended in 45 μl of the respective culture medium to which 5 μl (500 units) of β-glucuronidase was added. Following a 12 h incubation at 37°C, the reaction was terminated by the addition of methanol, after which the samples were treated as described above.

#### *In vivo* assessment of CYP induction

The *in vivo* characterization of the Basel cocktail has been described in previous publications (Donzelli et al., 2014; Derungs et al., 2016). Fifteen healthy volunteers were treated with 600 mg rifampicin per day for 7 days to induce CYP activity (Derungs et al., 2016). Genotyping identified one subject as a CYP2D6 intermediate metabolizer (CYP2D6^*^4/^*^41) and another subject as a CYP2C19 poor metabolizer (CYP2C19^*^2/^*^2). Data from these two subjects were not included in the analysis for CYP2C19 (*n* = 14) and CYP2D6 (*n* = 14), respectively. Clearance ratios of the probe drugs (induced vs. non-induced) were used to assess the extent of CYP induction.

#### Data analysis

CYP activities were determined as the respective metabolite formation rates corresponding to the slope in the metabolite concentration vs. time graphs. Metabolite concentrations were quantified using standard curves of pure compounds as previously described (Donzelli et al., 2014).

For induction experiments, formation rates were determined and the fold change vs. the basal conditions was calculated as the ratio of the metabolite formation rate in wells exposed to an inducer and control wells.

Means were compared with the two-tailed Student's *t*-test using GraphPad Prism 6.0 (GraphPad Software, San Diego, CA, USA). Data are presented as mean ± SEM unless stated otherwise.

## Results

CYP mRNA expression and CYP activities were investigated using four different liver cell cultures: HepG2 cells, HepaRG cells, PHHs seeded two-dimensionally (2D), and primary hepatocytes from the same donor in three-dimensional (3D) spheroid co-culture.

### Basal mRNA expression of CYPs in the different liver cell models

**Figure 1:**
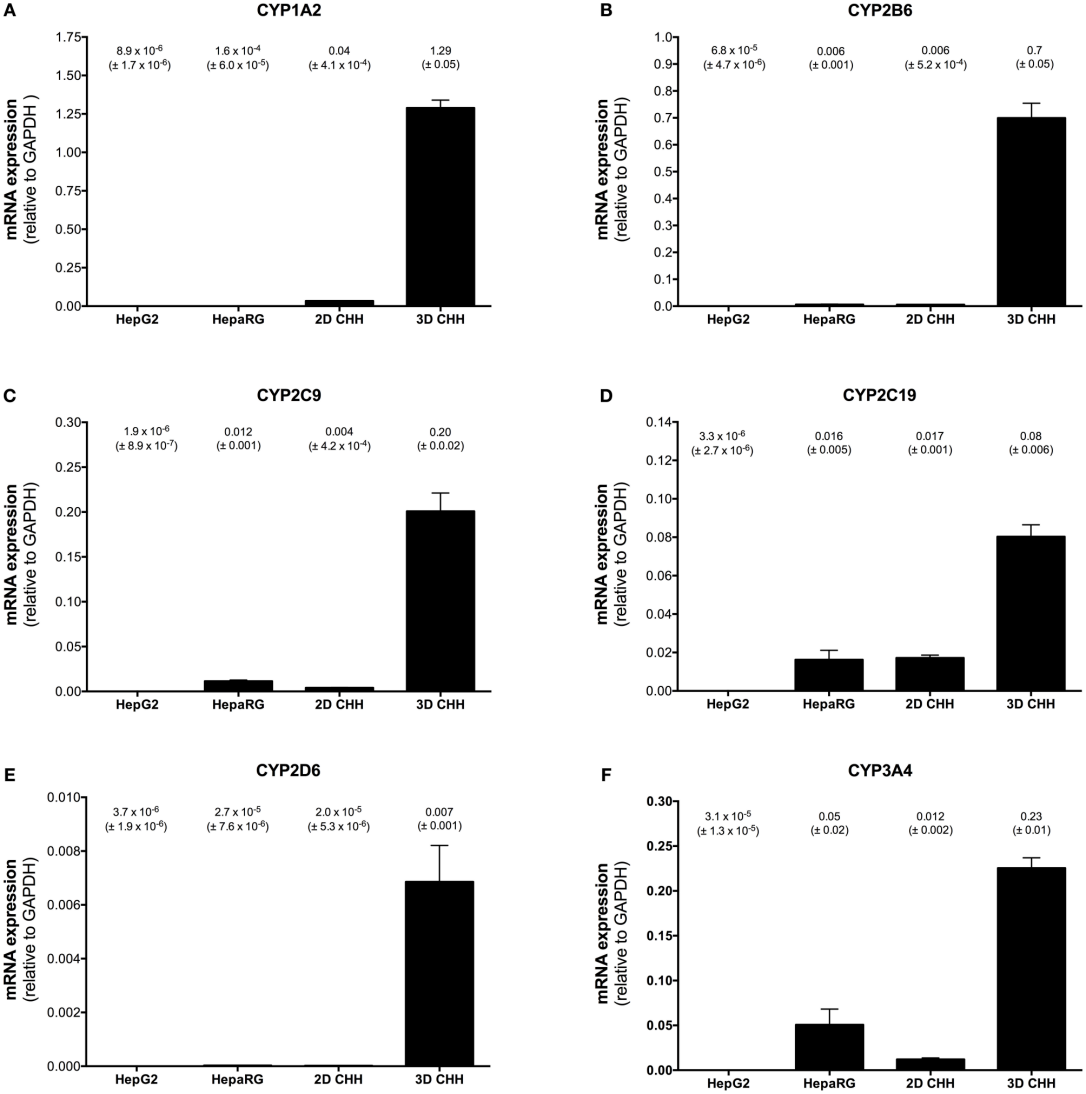
Basal mRNA expression of CYP1A2 **(A)**, 2B6 **(B)**, 2C9 **(C)**, 2C19 **(D)**, 2D6 **(E)**, and 3A4 **(F)** in HepG2, HepaRG, 2D-, and 3D-cultured primary cryopreserved human hepatocytes. mRNA expression was determined by rt-PCR as described in the Materials and Methods Section. Results are expressed as mean ± SEM relative to GAPDH of at least three independent experiments. PHH, primary human hepatocytes.

Basal CYP mRNA expression relative to GAPDH was detectable in all cell systems investigated and was highest in 3D-cultured PHHs (Figure 1). For HepG2 cells, the CYP mRNA expression was lower than for every other cell system investigated. In comparison to 3D-cultured PHHs, HepaRG cells showed a 4 times lower mRNA expression of CYP3A4 and 2C19, whereas the mRNA expression of the other CYPs investigated was more than 10 times lower. In 2D-cultured PHHs, CYP2C19 mRNA expression was ~4 times lower than in 3D-cultured hepatocytes, whereas the mRNA expression of the other CYPs was more than 10 times lower.

### Basal metabolic activity of the liver cell models used

Experiments using single substrates instead of the entire cocktail showed no mutual interactions between the probe drugs, except for 10 μM efavirenz, which was associated with a 30% decrease in CYP2C9 and a 20% decrease in CYP2C19 activity (results not shown). For HepaRG and conventional hepatocyte incubations, the use of 10 μM efavirenz was necessary, as lower efavirenz concentrations did not yield quantifiable concentrations of 8′-hydroxyefavirenz. All experiments were performed with the entire cocktail, hence the slight inhibitory effect of efavirenz was present in both conditions (basal and induced). In the *in vivo* cocktail, this interaction had been avoided by using efavirenz at a very low dose (Derungs et al., 2016).

Basal activities for all substrates investigated could be determined in HepaRG cells, 2D- and 3D-cultured PHHs, but not in HepG2 cells (Table 2). The highest activities were observed for CYP3A4, CYP2C19, and CYP2D6 in 3D-cultured PHHs and in HepaRG cells. In HepG2 cells, only activities of CYP3A4 and CYP2C19 could reliably be determined; metabolite formation remained below the limit of detection for the other CYPs.

**Table 2:** **Basal CYP activities**.

	**Basal CYP activities pmol/h/50,000 cells**			
	**HepG2 cells**	**HepaRG cells**	**2D-cultured PHH**	**3D-cultured PHH**
CYP1A2	nd	(0.46 ± 0.02)	(0.09 ± 0.01)	0.22 ± 0.02
				0.932
CYP2B6	nd	0.33 ± 0.02	0.54 ± 0.04	2.11 ± 0.07
		0.999	0.905	0.985
CYP2C9	nd	(0.11 ± 0.01)	(0.016 ± 0.002)	0.16 ± 0.01
				0.854
CYP2C19	0.16 ± 0.01	18.5 ± 1.0	0.47 ± 0.04	3.7 ± 0.1
	0.976	0.939	0.815	0.978
CYP2D6	nd	0.69 ± 0.06	0.25 ± 0.01	6.2 ± 0.3
		0.823	0.950	0.935
CYP3A4	0.64 ± 0.04	85.6 ± 4.5	27.4 ± 1.5	60.4 ± 1.5
	0.645	0.991	0.903	0.993

**Figure 2:**
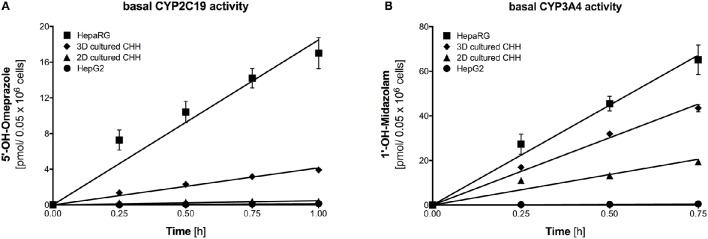
Basal activity of CYP2C19 and CYP3A4 assessed using formation rates of 5-hydroxyomeprazole **(A)** and 1′-hydroxymidazolam **(B)** in HepG2, HepaRG, 2D-, and 3D-cultured primary cryopreserved human hepatocytes. Primary hepatocytes were from the same batch of cryopreserved cells. Metabolite concentrations were determined by LC-MS/MS. The corresponding velocities and regressions are given in Table 2. Data are presented as mean ± SEM of at least three independent experiments. PHH, primary human hepatocytes.

*Basal CYP activity in HepG2, HepaRG, 2D-, and 3D-cultured primary cryopreserved human hepatocytes (PHH). Primary human hepatocytes used for 2D- and 3D-cultures were from the same batch of cryopreserved cells. Values presented are the metabolite formation rates (see Figure 2) and the corresponding regression coefficients (r^2^) of the metabolite-time curves. Metabolite concentrations were determined by LC-MS/MS. Data are presented as mean ± SEM of at least three independent experiments. Values in brackets are based on measurements of the last experimental time-point only, concentrations of all earlier time-points were below LLOQ. nd = not determinable (i.e., lacking basal activity)*.

Figure 2 shows the metabolite formation rates for the two CYP isoforms (CYP2C19 and CYP3A4) with activity in all four cell models using omeprazole and midazolam as probe drugs. Metabolite accumulation was linear with time up to 45 min for CYP3A4 and up to 1 h for CYP2C19, respectively. The metabolite formation rates were highest for HepaRG cells and lowest for HepG2 cells. Formation rates in 3D-cultured primary hepatocytes were higher than in 2D-cultures, illustrating a benefit of the 3D spheroid co-culture.

### Induced mRNA expression of CYPs in liver cell models exposed to rifampicin

**Figure 3:**
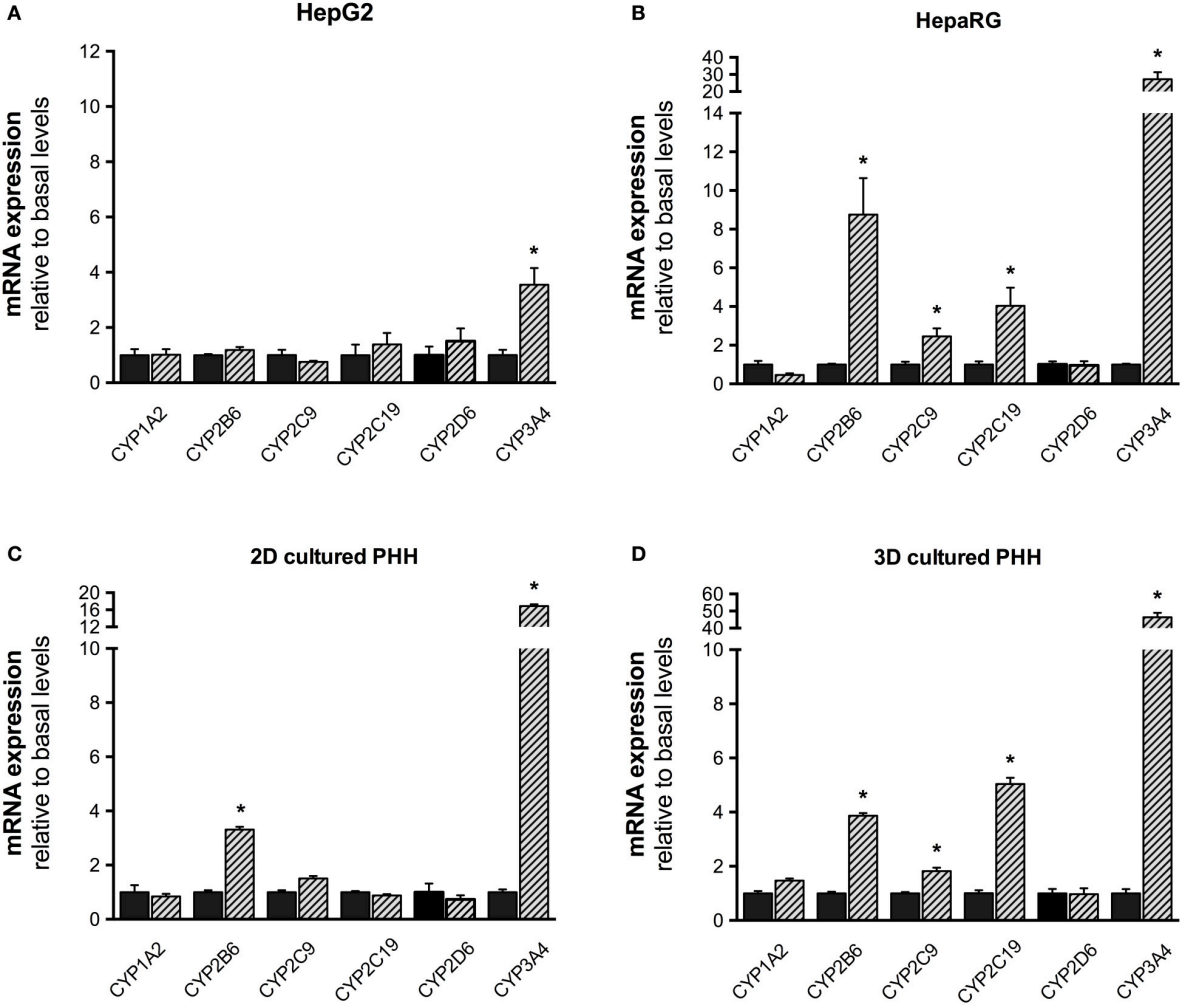
mRNA expression of CYP1A2, 2B6, 2C9, 2C19, 2D6, and 3A4 in HepG2 **(A)**, HepaRG **(B)**, 2D- **(C)**, and 3D-cultured primary cryopreserved human hepatocytes **(D)** after treatment with 20 μM rifampicin for 48 h. mRNA expression was determined by rt-PCR as described in the Materials and Methods Section. Values were normalized to GAPDH expression. Results are expressed as the mean ± SEM fold increase after treatment with rifampicin relative to levels observed in control cultures. PHH, primary human hepatocytes. ^*^p <0.05 vs. control culture.

Liver cell models were exposed to 20 μM rifampicin for 48 h and mRNA expression of the 6 CYPs isoforms was determined. In HepG2 cells, mRNA expression increased only for CYP3A4 (Figure 3). In HepaRG cells, mRNA expression increased for CYP2B6, 2C9, 2C19, and 3A4, but not for CYP1A2 and 2D6. A similar pattern of CYP induction was obtained for 3D-cultured PHHs, showing induction for CYP2B6, 2C9, 2C19, and 3A4, but not for CYP2D6 and 1A2. In comparison, when primary hepatocytes from the same batch were cultured conventionally (2D), induced mRNA expression could only be found for CYP3A4 and CYP2B6.

Since rifampicin is only a weak inducer of CYP1A2, we exposed 3D-cultured PHHs also to 3-methylcholanthrene, which is a well-established *in vitro* CYP1A2 inducer (Morel et al., 1990; Rodríguez-Antona et al., 2000). As shown in Supplementary Figure 1, in 3D-cultured PHHs CYP1A2 mRNA was induced ~13 times by 3-methylcholathrene, which was also reflected in an ~6 times higher production of paraxanthine from caffeine.

### CYP activity after treatment of liver cell models with rifampicin

**Figure 4:**
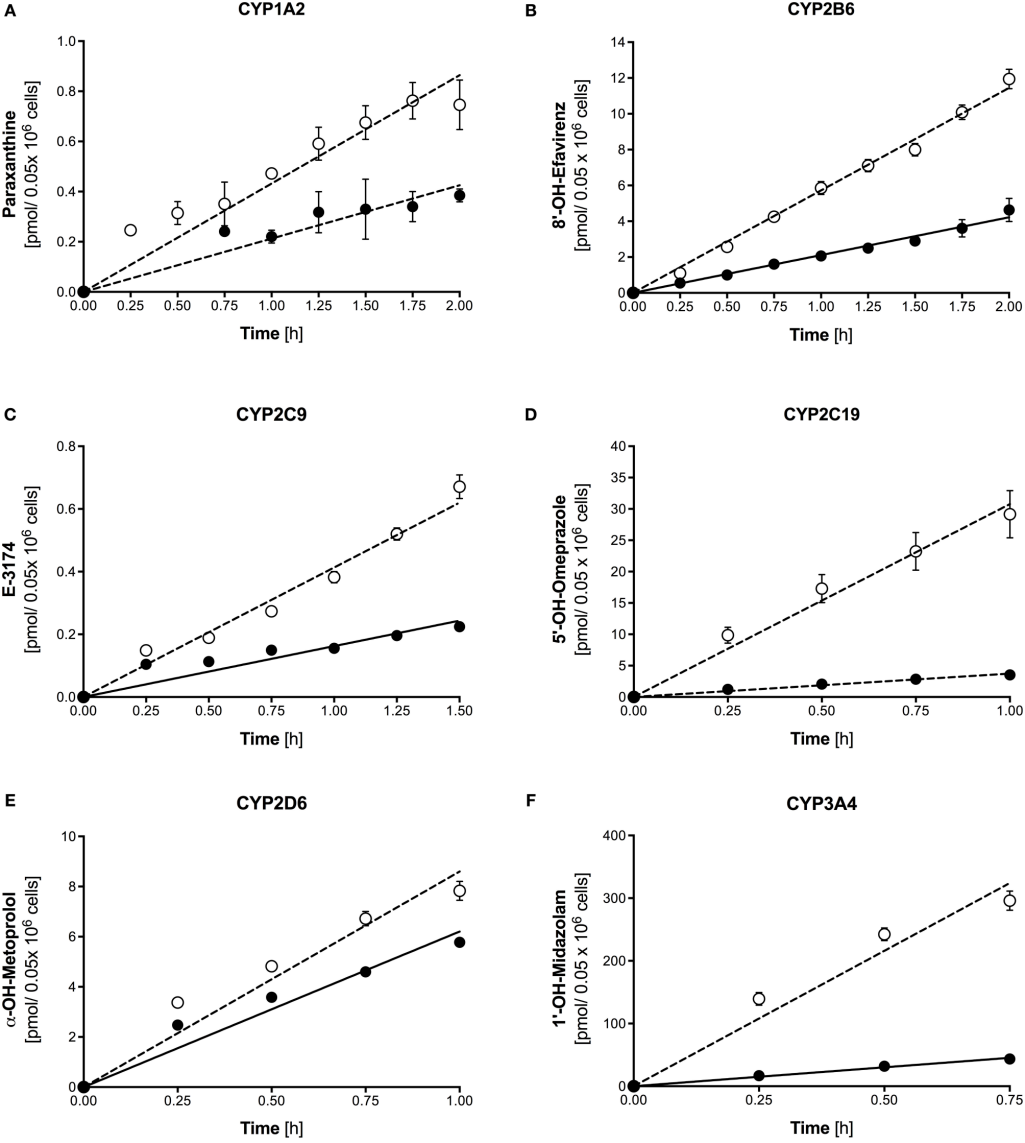
Formation of paraxanthine **(A)**, 8′-OH-efavirenz **(B)**, E-3174 **(C)**, 5-OH-omeprazole **(D)**, α-OH-metoprolol **(E)**, and 1′-hydroxymidazolam **(F)** by 3D-cultured primary human hepatocytes after treatment with 20 μM rifampicin for 72 h (open symbols) compared to control cultures (closed symbols). Metabolite concentrations were determined by LC-MS/MS. The corresponding formation rates and regression coefficients in control cultures and after treatment with 20 μM rifampicin are given in Tables 2, 3. Data are presented as mean ± SEM of at least three independent experiments.

Metabolite formation was assessed after treatment of liver cells with 20 μM rifampicin for 72 h. As shown in Figure 4, treatment of 3D-cultured PHHs was associated with increased metabolite formation by all tested CYP isoforms. The metabolite production rates were linear for at least 45 min (midazolam) and up to 120 min (caffeine and efavirenz).

In agreement with the mRNA expression data after induction with rifampicin and basal CYP activity, metabolite formation could be determined for all substrates in HepaRG cells, and in 2D- and 3D-cultured PHHs, but not in HepG2 cells (Table 3). The highest activities were detected for CYP3A4 and CYP2C19 in 3D-cultured PHHs and in HepaRG cells. In HepG2 cells, only activities of CYP3A4 and 2C19 could be detected.

**Table 3:** **Induced CYP activities**.

	**Induced CYP activities (20μM rifampicin) pmol/h/50,000 cells**			
	**HepG2 cells**	**HepaRG cells**	**2D-cultured PHH**	**3D-cultured PHH**
CYP1A2	nd	(0.51 ± 0.04)	(0.09 ± 0.01)	0.46 ± 0.02
				0.956
CYP2B6	nd	1.31 ± 0.04	1.7 ± 0.1	5.7 ± 0.1
		0.998	0.975	0.994
CYP2C9	nd	(0.12 ± 0.02)	(0.022 ± 0.002)	0.41 ± 0.01
				0.985
CYP2C19	0.21 ± 0.01	75.9 ± 1.8	1.96 ± 0.11	30.7 ± 1.7
	0.979	0.996	0.978	0.980
CYP2D6	nd	3.9 ± 0.2	0.35 ± 0.01	8.6 ± 0.4
		0.992	0.928	0.952
CYP3A4	1.1 ± 0.1	675 ± 20	129 ± 8	432 ± 17
	0.999	0.999	0.943	0.952

*CYP activity in HepG2, HepaRG, 2D-, and 3D-cultured primary cryopreserved human hepatocytes (PHH) after treatment with 20 μM rifampicin for 72 h. Primary human hepatocytes used for 2D- and 3D-cultures were from the same batch of cryopreserved cells. Values presented are the metabolite formation rates and the regression coefficients (r^2^) of the metabolite-time curves after pre-treatment with rifampicin. Metabolite concentrations were determined by LC/MS. Data are presented as mean ± SEM of at least three independent experiments. Values in brackets are based on measurements of the last experimental time-point only, concentrations of all earlier time-points were below LLOQ. nd = not determinable (lacking basal activity)*.

### Comparison rifampicin-induced CYP activity *in vitro* and *in vivo*

**Figure 5:**
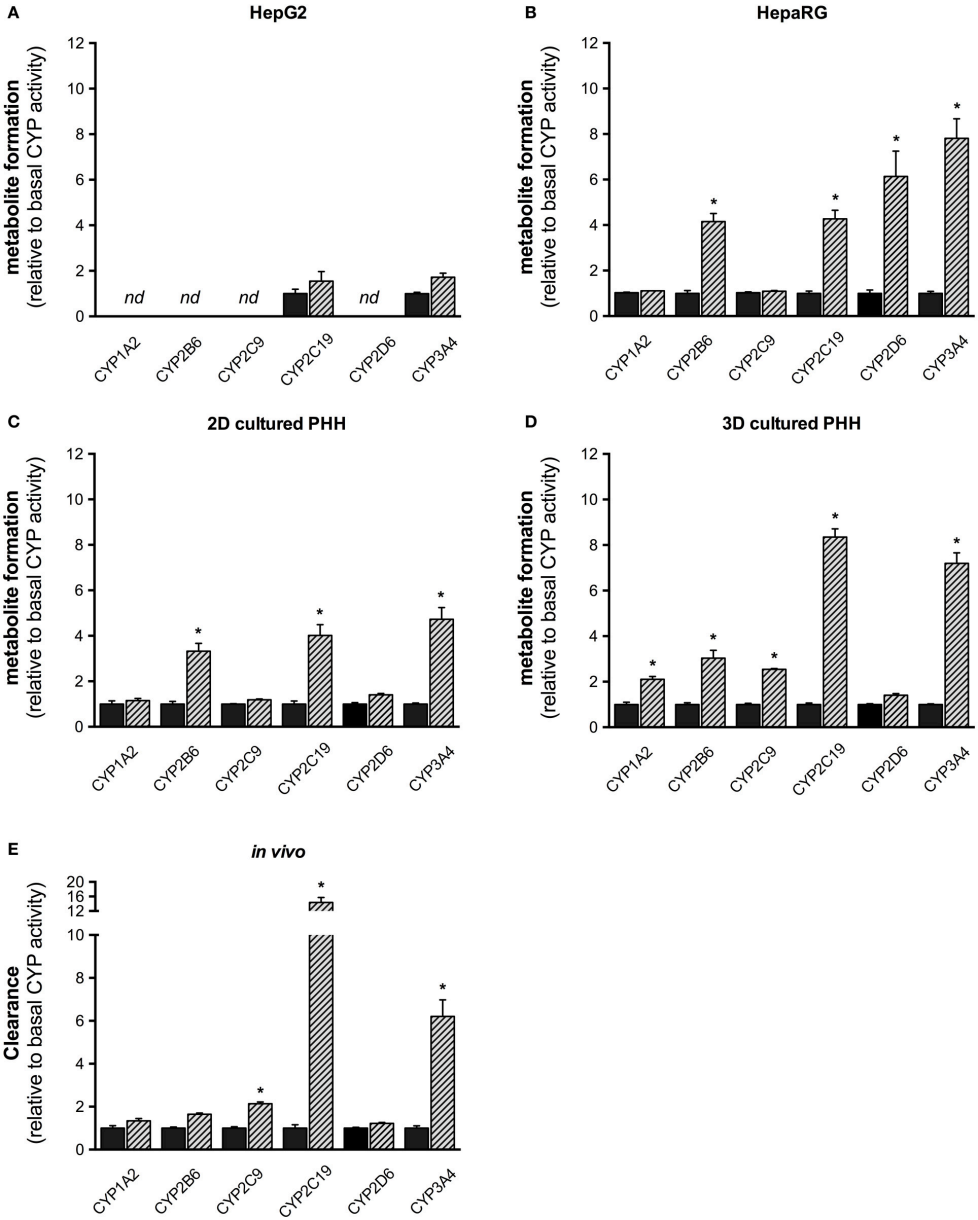
Fold increase (relative to non-induced control) of CYP activity *in vitro* in HepG2 **(A)**, HepaRG **(B)**, 2D-, and 3D-cultured primary human hepatocytes **(C,D)**, and *in vivo* in healthy male subjects (**E**, *n* = 14 for CYP2C19 and CYP2D6; *n* = 15 for all other CYP isoforms) after treatment with rifampicin. Hepatocyte cell lines were treated with 20 μM rifampicin for 72 h and healthy volunteers with 600 mg rifampicin per day for 7 days as described in the Materials and Methods Section. Metabolite and parent drug concentrations were determined by LC-MS/MS. Results are expressed as mean ± SEM. PHH, primary human hepatocytes. ^*^p <0.05 vs. control.

Compared to baseline, CYP2C9 and CYP3A4 activities were increased 1.5–2 times in HepG2 cells without reaching statistical significance (Figure 5). In HepaRG cells, activities of CYP3A4, CYP2D6, 2C19, and 2B6 were increased significantly, with the exception of CYP2D6 matching well with the mRNA induction data. An almost identical result was obtained for 2D-cultured PHHs. In 3D-cultured PHHs, CYP activities of all inducible CYP isoforms were increased at least two-fold after rifampicin induction.

Treatment of healthy volunteers with rifampicin (Derungs et al., 2016) was associated with an approximately seven-fold induction of CYP3A4, a 14-fold induction of CYP2C19, a doubling of CYP2C9 activity and an ~1.7-fold increase in CYP2B6 activity. When compared to the liver cell models investigated, this pattern was reflected best by the 3D-cultured PHHs.

## Discussion

This study confirms that basal mRNA expression and CYP activity as well as CYP inducibility show large differences between *in vitro* used hepatocyte cell models and, depending on the hepatocyte model used, that the *in vivo* characterized Basel phenotyping cocktail is suitable for CYP induction experiments *in vitro*.

HepG2 cells represent a hepatoma cell line, which is used extensively for toxicological studies but less frequently for studies of drug metabolism (Yoshitomi et al., 2001; Xu et al., 2004; Zahno et al., 2011). Drug metabolism is difficult to assess with this cell line since CYPs show weak mRNA expression and activity (Donato and Castell, 2003; Westerink and Schoonen, 2007a; Gerets et al., 2012). In this point, the results of the current investigation are in agreement with these previous studies. In the basal state, mRNA expression was clearly lower than in the other cell types for every CYP investigated and the activity could reliably be determined only for CYP3A4 and CYP2C19. CYP induction with rifampicin resulted in a significant four-fold increase in the CYP3A4 mRNA, which was only partially reflected in a corresponding increase in CYP3A4 activity. A limited inducibility of the CYP3A and CYP2C families in HepG2 cells has also been shown in other studies (Donato and Castell, 2003; Gerets et al., 2012) and has been attributed to a low expression of the nuclear receptors CAR and PXR (Aninat et al., 2006; Guillouzo et al., 2007), which are mediating the induction of the CYP2C and 3A families (Xu et al., 2005). In contrast, inducibility of CYP1A2 (which we did not determine in our studies) has been shown to be maintained in HepG2 cells (Choi et al., 2015). CYP1A2 induction is mediated by the aryl hydrocarbon receptor (Xu et al., 2005), which is highly expressed in HepG2 cells (Aninat et al., 2006). In contrast to phase I enzyme activities, conjugation reactions appear to be maintained quite well in HepG2 cells (Westerink and Schoonen, 2007b). HepG2 cells can therefore only be used to answer very specific questions regarding drug metabolism.

HepaRG cells represent a hepatoma cell line, which has first been described in 2002 as a cell model suitable for HBV infection and propagation (Gripon et al., 2002). Soon after their first description, HepaRG cells were characterized and proposed for their use as a model for drug metabolism studies (Aninat et al., 2006). The gene expression profile shows profound differences compared to HepG2 cells, explaining the observed differences in their drug metabolism phenotype (Jennen et al., 2010). HepaRG cells have also been used in drug toxicity studies, particularly, when active metabolites are suspected (Guillouzo et al., 2007; Gerets et al., 2012). Considering the current study, HepaRG cells were clearly a more suitable hepatocyte model for studying drug metabolism than HepG2 cells. mRNA expression could be detected for all CYPs investigated and was inducible except for CYP1A2 and 2D6. Lack of CYP1A2 induction is not a problem of the cell line, but of rifampicin, which was used as a CYP inducer. As shown *in vitro* (Gerets et al., 2012; Choi et al., 2015) and *in vivo* (Derungs et al., 2016), rifampicin is a good inducer for the CYP2C and 3A families, but not for CYP1A2. With specific CYP1A2 inducers such as 3-methylcholanthrene CYP1A2 can be induced in HepaRG cells on both the mRNA and the activity level (Aninat et al., 2006; Guillouzo et al., 2007). CYP2D6 has a low activity in HepaRG cells, possibly because the cell line originates from a slow CYP2D6 metabolizer (Guillouzo et al., 2007), and it cannot be induced with rifampicin (Gerets et al., 2012).

Interestingly, the activity for CYP3A4 and 2C19 were higher in HepaRG cells as compared to 3D-cultured PHHs, whereas the corresponding mRNA expressions (relative to GAPDH) were lower. This could be a problem of standardization to GAPDH (different GAPDH expression in HepaRG cells and primary hepatocytes) or a bad correlation between mRNA levels and protein expression and/or activity. In the study of Choi et al. (2015), GAPDH mRNA expression was quite stable among different hepatocyte cell lines, arguing against the first possibility. On the other hand, it is well established that posttranslational modifications such as acetylation, glycosylation, and phosphorylation can have a large impact on protein activity (Glanemann et al., 2003), favoring the second possibility. In our study, the CYP activity profile and inducibility of HepaRG cells were more closely related to primary hepatocytes than to HepG2 cells.

Primary hepatocytes are currently considered to be the gold standard for drug metabolism studies. By using the same batch of human cryopreserved hepatocytes in the 2D- and 3D-cultures, we were able to estimate whether the 3D environment would lead to differences in CYP activity and/or expression compared to the 2D-cultures. Indeed, in comparison to 2D-cultures, 3D-cultured PHHs exhibited a higher CYP mRNA expression, a higher basal CYP activity and a better inducibility. In comparison to the *in vivo* results regarding CYP induction, 3D-cultured PHHs showed a better agreement than the other cell lines tested, suggesting that they could be usable to predict the induction potential of new drugs. Hepatocyte 3D-cultures are currently being developed and characterized as an alternative to 2D-cultures in order to overcome well-known drawbacks of 2D-cultures such as varying expression of CYP activities, varying CYP inducibility and loss of differentiation over time (Aninat et al., 2006; Halladay et al., 2012). Assuming that 3D-cultures of PHHs may offer advantages compared to the corresponding 2D-cultures, many 3D-hepatocyte models have been and are currently being explored for drug metabolism and drug toxicity studies (Godoy et al., 2013; Schyschka et al., 2013; Astashkina and Grainger, 2014; Roth and Singer, 2014).

We performed our studies with a co-culture system using 3T3-Swiss albino mouse fibroblasts as feeder cells for human cryopreserved primary hepatocytes. This system has originally been described in detail by Ohkura et al. (2014) who characterized it regarding the mRNA expression of phase I and phase II drug metabolism enzymes and demonstrated its capacity to produce metabolites from different drugs. The model uses special plates coated with a block co-polymer that allows the fibroblasts and hepatocytes to only adhere to certain areas of the wells. In time, the mouse fibroblasts and human cryopreserved hepatocytes are able to form 3D structures described as spheroids. Spheroids can be regarded as a cell aggregation with an energy- and surface-minimized structure that, according to van Zijl et al. (van Zijl and Mikulits, 2010) mimic the *in vivo* situation quite efficiently regarding cell shape and cellular environment. The results of our study support this assumption.

After having been characterized *in vivo* (Donzelli et al., 2014; Derungs et al., 2016), the substrate cocktail showed a satisfactory performance also *in vitro*. Critical substrates were losartan (CYP2C9) and caffeine (CYP1A2), which were metabolized at a low rate even in 3D-cultured PHHs and showed no significant metabolism in HepG2 cells. On the other hand, they correctly reflected CYP2C9 induction by rifampicin and CYP1A2 induction by 3-methylcholanthrene. Depending on the question to be answered and on the cell system used, they could be replaced by phenacetin (CYP1A2) and diclofenac (CYP2C9), which have been shown to be suitable substrates *in vitro* (Youdim et al., 2007; Mori et al., 2009; Halladay et al., 2012).

In conclusion, our studies demonstrate that the 3D-PHH spheroid culture system is suitable for the assessment of drug metabolism and CYP induction *in vitro*. Among the four tested cell systems, 3D-cultured PHHs best reflect CYP inducibility *in vivo*. HepaRG cells are close to 3D-cultured PHHs but induced CYP activities correlate less well with CYP induction *in vivo*. HepG2 cells are known to predict CYP1A2 induction but are less suitable for CYPs of the 2C and 3A family. The substrates of the Basel phenotyping cocktail only show minimal interactions *in vitro* and the cocktail is thus usable for the assessment of CYP induction *in vitro*.

## Author contributions

BB performed research, interpreted data, and wrote manuscript. MD performed research, interpreted data. SM provided research tools. FB provided research tools, interpreted data. AR provided research tools, interpreted data. SK designed research, interpreted data, wrote manuscript. MH designed research, interpreted data, wrote manuscript.

## Funding

SK was supported by a grant of the Swiss National Science Foundation (SNF 31003A_156270).

### Conflict of interest statement

The authors declare that the research was conducted in the absence of any commercial or financial relationships that could be construed as a potential conflict of interest.

## Supplementary material

Supplementary Figure 1 **(A)** Activity of CYP1A2 assessed using caffeine-N_3_-demethylation in 3D-cultured primary cryopreserved human hepatocytes after treatment with 3-methylcholanthrene (2 μM; open symbols) for 72 h compared to control cultures (closed symbols). **(B)** Fold increase (relative to non-induced control) of CYP1A2 mRNA levels after treatment of 3D-cultured primary human hepatocytes with 3-methylcholanthrene (2 μM) for 72 h. mRNA levels were determined using rtPCR as described in the Materials and Methods Section. mRNA expression in control cultures and after treatment with 3-methylcholanthrene was first normalized to the respective GAPDH expression. Data are given as mean ± SEM of at least three independent experiments. ^*^*p* <0.05 vs. control.

Supplementary Figure 2 **Chemical structures of the probe drugs and their phase I metabolites of the Basel phenotyping cocktail**.
