# Supplementary figures and images for: Comparison of Liver Cell Models Using the Basel Phenotyping Cocktail
Source: Front Pharmacol. 2016 Nov 21;7:443. doi: 10.3389/fphar.2016.00443 (PMC5116554; doi:10.3389/fphar.2016.00443)

Suppl. Fig. 1

- basal (DMSO / 0.1% v/v)
- ◇ induced (3-Methylcholanthrene/ 2 $\mu$ M)

A

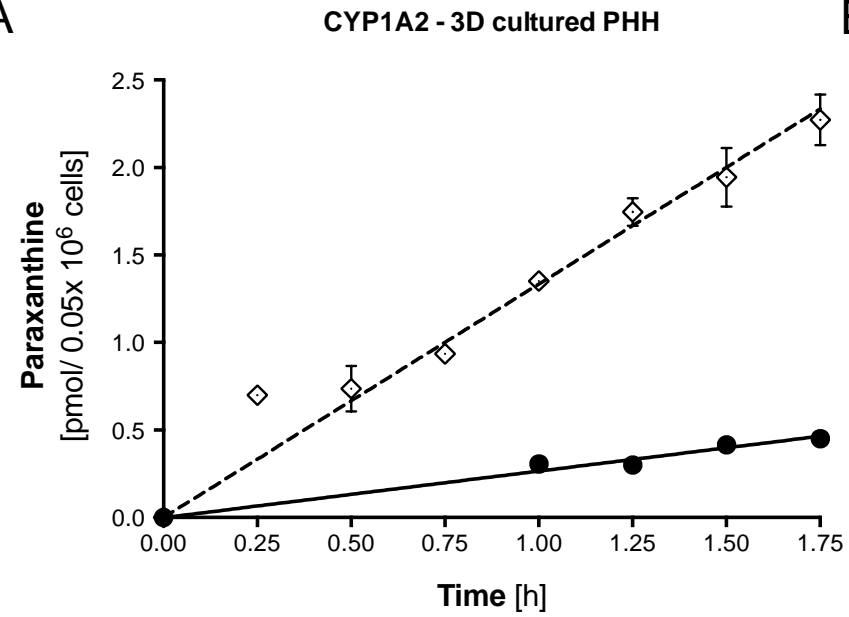

B

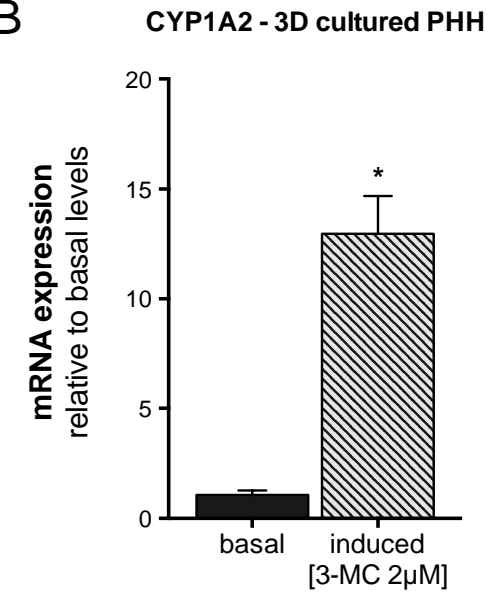

Supplement: Supplementary Figure 1 — (A) Activity of CYP1A2 assessed using caffeine-N3-demethylation in 3D-cultured primary cryopreserved human hepatocytes after treatment with 3-methylcholanthrene (2 μM; open symbols) for 72 h compared to control cultures (closed symbols). (B) Fold increase (relative to non-induced control) of CYP1A2 mRNA levels after treatment of 3D-cultured primary human hepatocytes with 3-methylcholanthrene (2 μM) for 72 h. mRNA levels were determined using rtPCR as described in the Materials and Methods Section. mRNA expression in control cultures and after treatment with 3-methylcholanthrene was first normalized to the respective GAPDH expression. Data are given as mean ± SEM of at least three independent experiments. *p <0.05 vs. control. [file Image1.PDF]
