# Supplementary material for: Comparison of Liver Cell Models Using the Basel Phenotyping Cocktail
Source: Front Pharmacol. 2016 Nov 21;7:443. doi: 10.3389/fphar.2016.00443 (PMC5116554; doi:10.3389/fphar.2016.00443)
Supplement: Supplementary Figure 2 — Chemical structures of the probe drugs and their phase I metabolites of the Basel phenotyping cocktail. [file Image2.PDF]

Supplementary Figure 2: Chemical structures of the probe drugs and their phase I metabolites of the Basel phenotyping cocktail

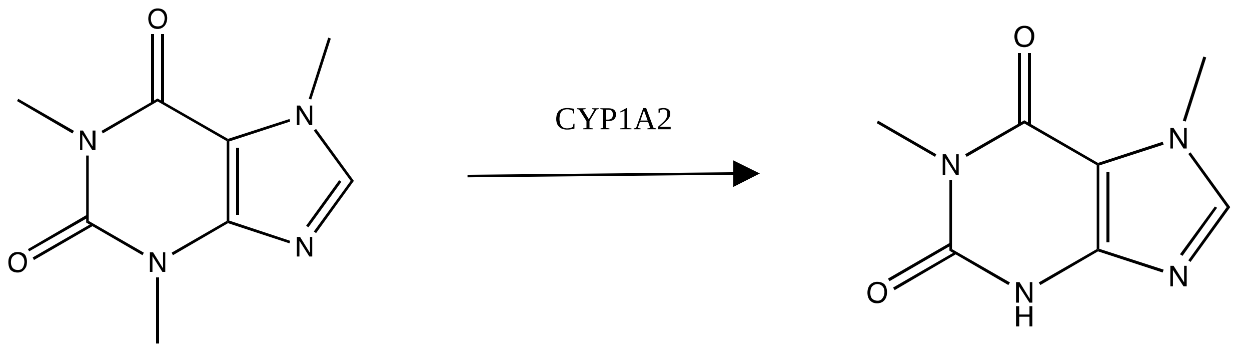

caffeine paraxanthine

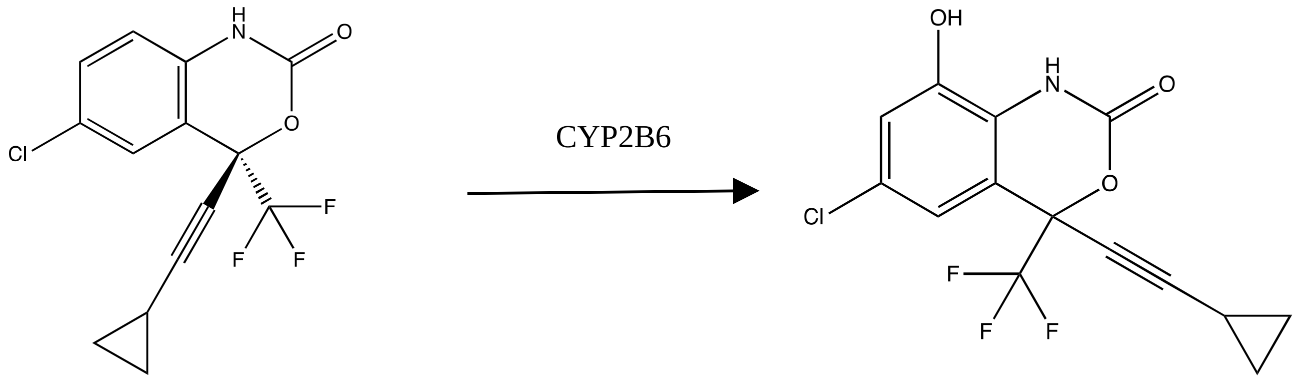

efavirenz 8’-hydroxyefavirenz

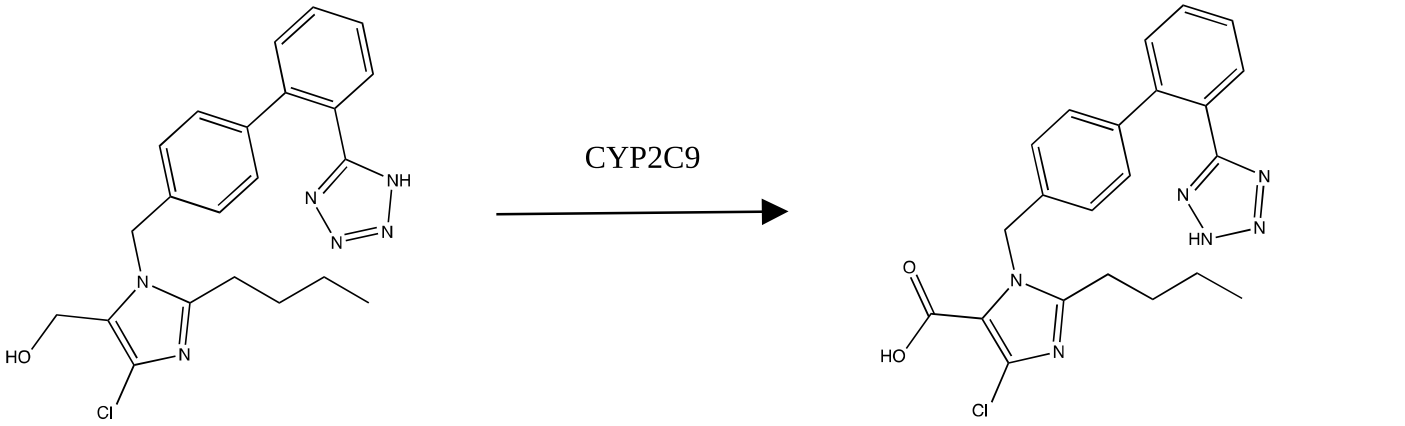

losartan E-3174

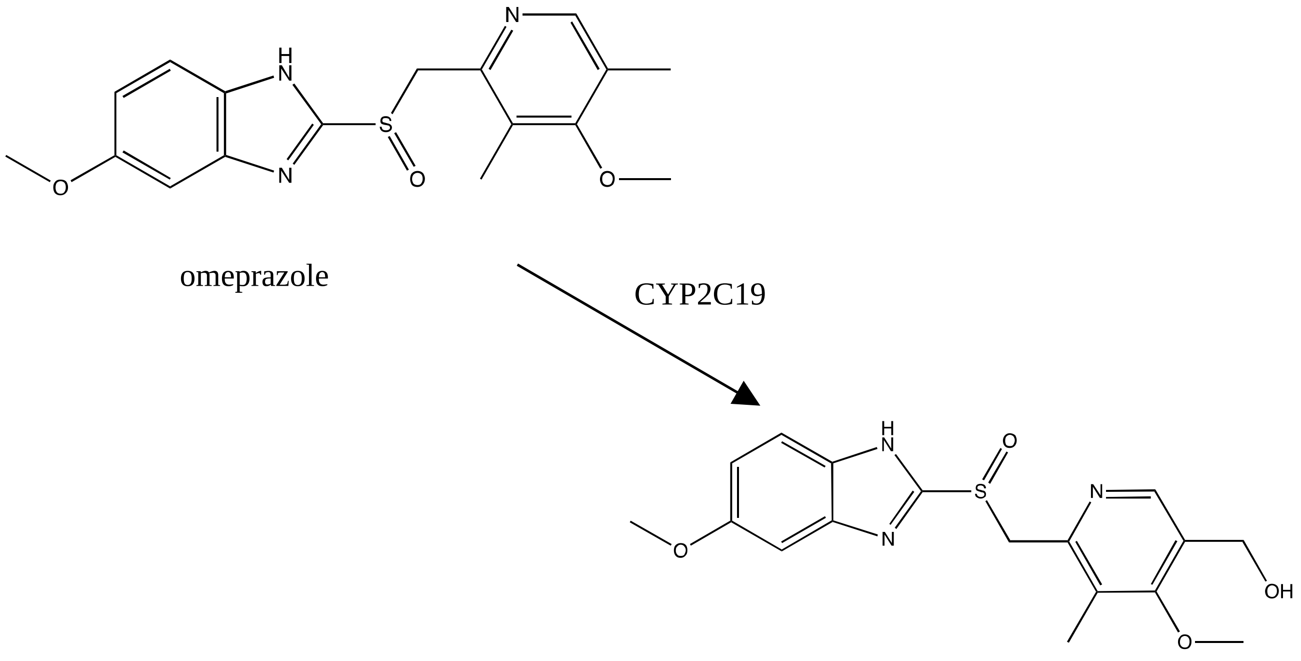

5’-hydroxyomeprazole

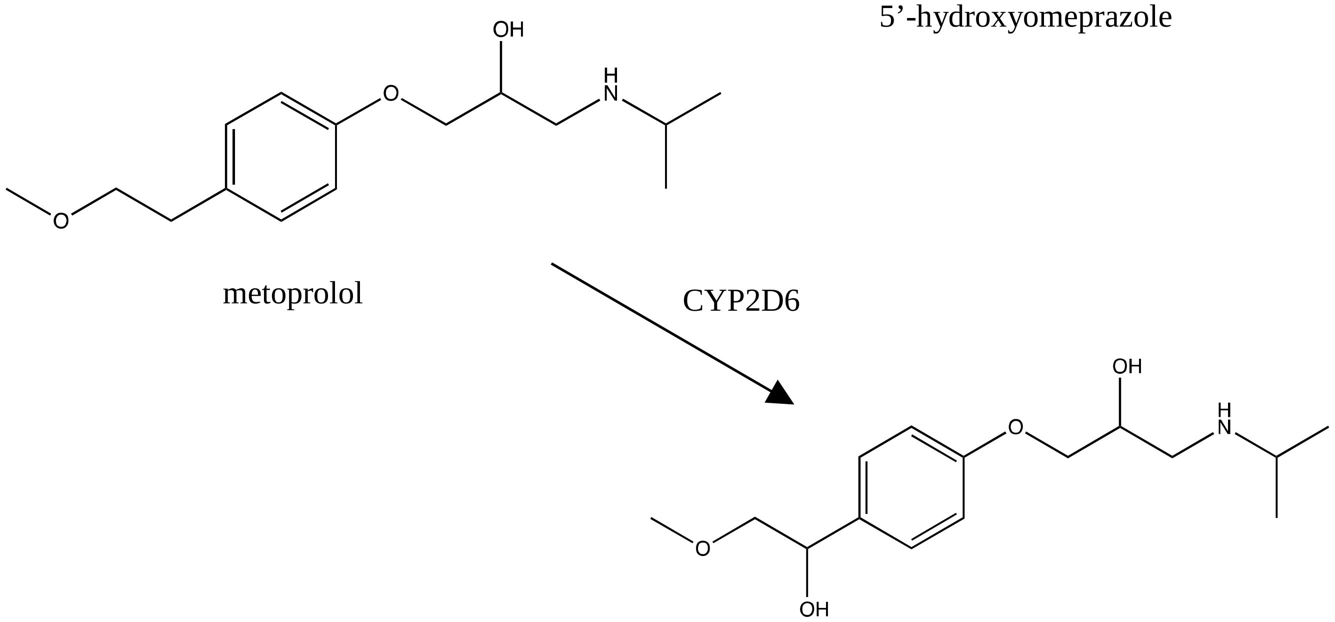

α-hydroxymetoprolol

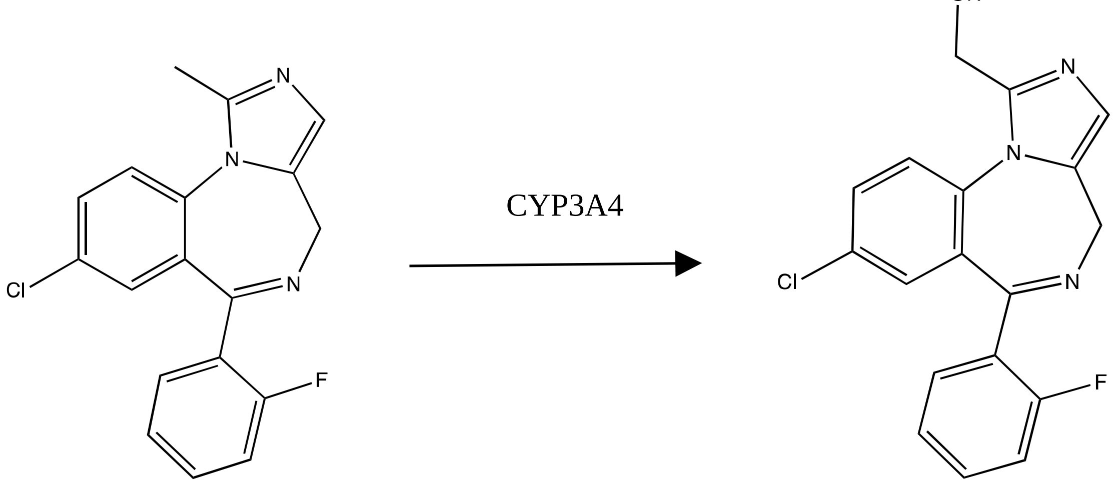

midazolam

1’-hydroxymidazolam
